# Enhanced and Proficient Soft Template Array of Polyaniline—TiO_2_ Nanocomposites Fibers Prepared Using Anionic Surfactant for Fuel Cell Hydrogen Storage

**DOI:** 10.3390/polym15204186

**Published:** 2023-10-22

**Authors:** Nacer Badi, Aashis S. Roy, Hatem A. Al-Aoh, Mohamed S. Motawea, Saleh A. Alghamdi, Abdulrhman M. Alsharari, Abdulrahman S. Albaqami, Alex Ignatiev

**Affiliations:** 1 Department of Physics, Faculty of Science, University of Tabuk, Tabuk 71491, Saudi Arabia; saalghamdi@ut.edu.sa (S.A.A.); aalsharari@ut.edu.sa (A.M.A.); asb91asb@gmail.com (A.S.A.); 2 Renewable Energy and Environmental Technologies Research Center, University of Tabuk, Tabuk 71491, Saudi Arabia; 3 Department of Chemistry, S.S. Tegnoor Degree College, Gubbi Colony, Kalaburagi 585105, Karnataka, India; aashisroy@gmail.com; 4 Department of Chemistry, Faculty of Science, University of Tabuk, Tabuk 71491, Saudi Arabia; 5 Department of Physics, University of Houston, Houston, TX 77204-5004, USA; ignatiev@uh.edu

**Keywords:** polyaniline fiber, hydrogen storage, surfactant, nanocomposites, DC conductivity

## Abstract

Porous TiO_2_-doped polyaniline and polyaniline nanocomposite fibers prepared by the in situ polymerization technique using anionic surfactant in an ice bath were studied. The prepared nanocomposites were characterized by FTIR spectroscopy and XRD patterns for structural analysis. The surface morphology of the polyaniline and its nanocomposites was examined using SEM images. DC conductivity shows the three levels of conductivity inherent in a semiconductor. Among the nanocomposites, the maximum DC conductivity is 5.6 S/cm for 3 wt.% polyaniline-TiO_2_ nanocomposite. Cyclic voltammetry shows the properties of PANI due to the redox peaks of 0.93 V and 0.24 V. Both peaks are due to the redox transition of PANI from the semiconductor to the conductive state. The hydrogen absorption capacity is approximately 4.5 wt.%, but at 60 °C the capacity doubles to approximately 7.3 wt.%. Conversely, 3 wt.% PANI—TiO_2_ nanocomposites have a high absorption capacity of 10.4 wt.% compared to other nanocomposites. An overall desorption capacity of 10.4 wt.% reduced to 96% was found for 3 wt.% TiO_2_-doped PANI nanocomposites.

## 1. Introduction

The rapid increase in risk from global warming has raised concerns among scientists and policymakers worldwide. Hydrogen fuel is a very important constituent in reducing global warming and greenhouse gas emissions [1]. Hydrogen is an almost zero carbon energy transporter and has the future potential to replace the fossil fuel used in a wide range of applications where conventional decarbonization techniques have demonstrated difficulties, such as heavy manufacturing industries and long-distance transportation. In fact, developed countries have recently declared their intentions to increase clean hydrogen production and consumption, with major global investment in hydrogen development predicted by 2030 [2,3,4]. Despite this, discussions and analyses of hydrogen’s usefulness in decreasing emissions have mainly ignored the warming impacts of hydrogen discharged into the atmosphere [5].

However, hydrogen is identified as a climate-friendly fuel that can power industries, buildings, ships, and planes without emitting carbon dioxide, making it an essential part of the transition to a low-carbon global economy and meeting net-zero greenhouse gas emission targets [6]. However, attention must be paid to avoiding hydrogen leakage, as this is critical to maximizing the fuel’s climatic benefits, regardless of how it is created [7]. While hydrogen has significant climate impacts that are often overlooked and underestimated, including the potential for more warming than perceived from hydrogen leakage, clean hydrogen production methods can greatly reduce greenhouse gas emissions, with blue and green hydrogen both having benefits and drawbacks depending on leakage rates and time horizons [8,9]. Finally, hydrogen fuel has the potential to considerably reduce global warming and greenhouse gas emissions; however, considerable consideration must be given to manufacturing methods and emission reductions in order to optimize its climatic benefits [6,10,11]. 

Hydrogen is considered the ideal fuel for the future since it dramatically reduces greenhouse gas emissions, decreases global reliance on fossil fuels, and improves the efficiency of the energy conversion process for both internal combustion engines and proton exchange membrane fuel cells [12,13]. Hydrogen storage affects both hydrogen generation and hydrogen applications, and so plays an important role in launching a hydrogen economy. Onboard hydrogen storage is an unavoidable requirement for today’s fuel cell vehicles and an indispensable component of the system that must be restructured [14]. Solid- state nanomaterials such as nano-semiconductors, nanometals, nanowires, nanotubes, and nanoporous and hollow materials have been used in energy and environmental applications in recent years due to their high hydrogen absorption or adsorption properties via various mechanisms [15]. When compared to physical hydrogen storage methods, these solid-state storage technologies have several advantages, including small material size, high efficiency, low weight, and low cost [16,17,18]. These physical storage solutions have some disadvantages, such as high costs and limited storage efficiency. 

Porous metal oxides (NiO, ZnO, Al_2_O_3_, MgO, and TiO_2_) have recently been investigated for hydrogen storage [19]. The important characteristics of TiO_2_, such as non-toxicity, chemical inertness, low cost, and environmental friendliness, make them suitable for use in a variety of applications such as paints, fuel cells, solar cells, gas sensors, hydrogen storage, antimicrobial materials, and photocatalysis [20,21,22]. There are materials that store hydrogen as hydrides and others that adsorb hydrogen molecules onto their surfaces. In general, hydrides act at high temperatures, but physisorbents require low temperatures to produce significant hydrogen sorption quantities [23]. 

Polyaniline fiber nanostructure has emerged as a promising material for hydrogen storage due to its high surface area and excellent conductivity. Its unique morphology allows for efficient adsorption and desorption of hydrogen molecules, making it an ideal candidate for fuel cell applications. Furthermore, the tunable properties of polyaniline fibers, such as their porosity and surface chemistry, can be tailored to enhance hydrogen storage capacity and improve overall performance [24]. Metal oxide in polyaniline nanocomposites plays a crucial role in enhancing the overall performance and properties of the material. The incorporation of metal oxide nanoparticles improves the conductivity, stability, and mechanical strength of polyaniline, making it suitable for various applications such as energy storage devices, sensors, and catalysis [25,26,27,28]. Additionally, metal oxide nanoparticles also provide a large surface area for better interaction with the surrounding environment, leading to improved sensing capabilities and enhanced reactivity [29,30]. Therefore, we made an attempt to prepare TiO_2_-doped polyaniline nanocomposite fiber using anionic surfactant. The prepared nanocomposites were subjected to characterization by Fourier transform infrared spectroscopy (FTIR) and X-ray diffraction pattern (XRD) for structural analysis and to surface morphology study using scanning electron microscopy (SEM) techniques. The transport studies were carried out by DC conductivity using a two-probe method and by AC conductivity using an impedance analyzer. The hydrogen sorption characteristics of the polyaniline and its nanocomposites were subjected to hydrogen absorption analysis by employing Hy-Energy’s PCTPro 2000 sorption equipment (SETARAM, Inc. St. Cisco, TX, USA). 

## 2. Materials and Methods

All the chemicals used in the preparation of the polyaniline and TiO_2_ nanoparticles were of analytical grade. Sulphuric acid (H_2_SO_4_) with a purity of 98.08%, titanium acetate anhydride (Ti(CH_3_COO)_2_(H_2_O)) with a purity of 99%, ammonium lauryl sulfate with a purity of 99%, orthophosphoric acid (H_3_PO_4_) with 99.99% purity, hydrogen peroxide (H_2_O_2_) with 99.99% purity with mol.wt. 34.01, 36.46% of concentrated hydrochloric acid, Toluene-4-sulfonic acid monohydrate (C_7_H_8_O_3_S. × H_2_O) with mol.wt. 172.20 and 98.6% purity, ammonium persulfate ((NH_4_)_2_S_2_O_8_) with 99.9% purity, polystyrene with Mw 350,000 and 99.9% purity, camphorsulfonic acid (C_10_H_16_O_4_S) with 99.8% purity, and acetone with 99.8% purity were procured from Sigma—Aldrich Pvt Lt, Gulbarga Karnataka, India, and used in their original states.

### 2.1. Synthesis of Porous TiO_2_ Nanoparticles

The synthesis of TiO_2_ nanoparticles by means of the sol–gel technique was as follows: 4.5 g of titanium acetate anhydride (Ti(CH_3_COO)_2_(H_2_O)) was dissolved in 100 mL ethanol and 4.5 g of sodium hydroxide (NaOH) was dissolved in 200 mL ethanol in a round-bottom flask. First, the solution of titanium acetate was mixed in a 500 mL beaker with continuous stirring for 20 min. Later, the solution of sodium hydroxide was added dropwise to the above solution, followed by hydrogen peroxide (H_2_O_2_), with continuous stirring at normal temperatures for 3 h, and the pH was maintained at 5.6 to 5.9. The pH was adjusted using less than 8.0% ethanol to allow settling of the white colloidal solution for 5 h. The white colloidal solution was transferred into centrifuge tubes and centrifuged at 10,000 rpm for 15 min, the supernatant liquid was discarded, and the white residue was washed with distilled water five to six times. The collected residue was dried at 60 °C for 60 min and the residue was annealed at 450 °C for 2 h in a temperature-controlled muffle furnace; we then crushed the annealed residue of the TiO_2_ nanoparticles into fine particles for use in the nanocomposite preparation [31].

### 2.2. Synthesis of Polyaniline Nanofibers 

To create an aniline hydrochloro-toluene-4-sulfonic acid solution, 100 mL aqueous solution of aniline (0.1 M) was combined with 100 mL of 1 M HCl, 100 mL of ammonium lauryl sulfate and 100 mL of a 1 M toluene-4-sulfonic acid monohydrate (PTSA) solution while constantly stirring. The mixture was then placed in an ice bath to soak the ice pores. Subsequently, 100 mL of ammonium persulfate (APS), 0.25 M, was gradually added while being continuously shaken at ice temperature. Once all the APS was added, the reaction mixture was placed in a deep freezer and allowed to react steadily for 24 h without stirring to produce a greenish spongy precipitate. The precipitate was filtered, repeatedly washed with distilled water to remove salt deposits, and then washed with 0.1 M HCl to remove unreacted monomers. To attain a consistent weight, the precipitate was dried under a dynamic vacuum for 48 h at 50 °C after being rinsed with acetone to remove the water from the nanocomposite [32,33,34].

### 2.3. Synthesis of Polyaniline–TiO_2_ Nanocomposite Fibers 

**Scheme 1:**
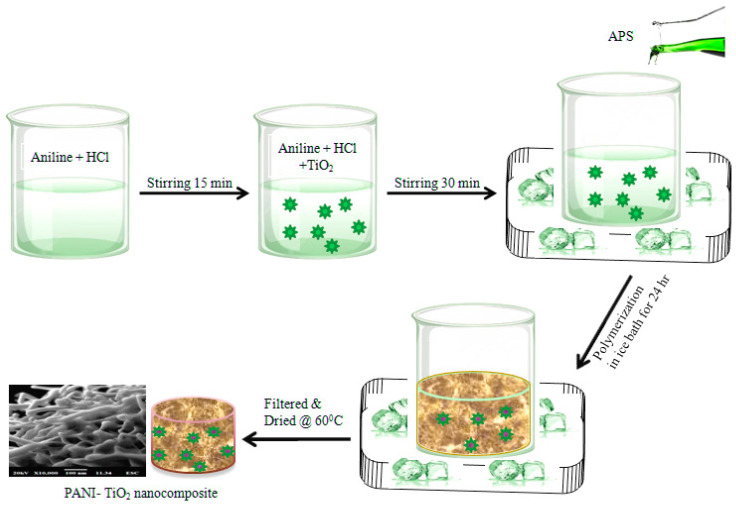
Schematic diagram of PANI—TiO_2_ nanocomposite synthesis.

The nanocomposites of the TiO_2_-doped polyaniline were prepared in a similar manner to the polyaniline synthesis. In this preparation process, aniline hydrochloro-toluene-4-sulfonic acid solution and 100 mL of ammonium lauryl sulfate were mixed with different weight percentages (1, 3, and 5 wt.%) of TiO_2_ nanoparticles with continuous stirring for 30 min. Subsequently, 100 mL of ammonium persulfate (APS), 0.25 M, was gradually added while continuously shaking at ice temperature. Once all the APS had been added, the reaction mixture was placed in the deep freezer and allowed to react steadily for 24 h without being stirred to produce a greenish spongy precipitate. The precipitate was filtered, repeatedly washed with distilled water to remove salt deposits, and then washed with 0.1 M HCl to get rid of unreacted monomers. In order to attain consistent weight, the precipitate was then dried under a dynamic vacuum for 48 h at 50 °C after being rinsed with acetone to remove the water from the nanocomposite [35]. The preparation of the PANI—TiO_2_ nanocomposite is represented in Scheme 1 below. 

## 3. Characterization Techniques

The Fourier transform infrared (FTIR) spectra of the polyaniline and PANI—TiO_2_ nanocomposite were recorded using a Perkin Elmer 1600 spectrophotometer, in the wave number range 400–4600 cm^−1^. The prepared samples were mixed with KBr at a ratio of 1:5 until a homogenous paste was formed, which was then put through a hydraulic press to form a 10 mm plate. The surface morphology of polyaniline and PANI—TiO_2_ nanocomposites that had been powder-coated with gold particles by sputtering was studied on an Au substrate. The CH Instruments 660D electrochemical workstation is used for electrochemical experiments, and a pico amp booster was used to detect the current and voltage. Electrochemical studies were performed using a three-electrode system. The first working electrodes were fabricated by coating polyaniline and its nanocomposite, a platinum wire counter electrode, and a saturated calomel electrode (SCE) as the reference electrode. 

The transport studies were carried out by DC conductivity by a two-probe method using Keithley Source Meter 2400, Cleveland, OH, USA. An electrolysis cell, a potentiostat, a current-to-voltage converter, and a data-gathering system make up a CV system. A working electrode, a counter electrode, a reference electrode, and an electrolytic solution make up the electrolysis cell. Cyclic Voltammetry (CV) is the most widely used electrochemical technique due to its ability to determine reaction processes using relatively low-cost equipment and speedy conducting tests. A general purpose potentiostat, the Gamry Interface 1010B, was used to carry out the cyclic voltammetry. The hydrogen sorption characteristics of polyaniline and its nanocomposites were subjected to hydrogen absorption analysis by employing Hy-Energy’s PCTPro 2000 sorption equipment. The HyDataV2.1 Lab-View program was used to examine the software subroutines for hydrogen purging cycles.

## 4. Results and Discussion

### 4.1. FTIR Spectra

**Figure 1:**
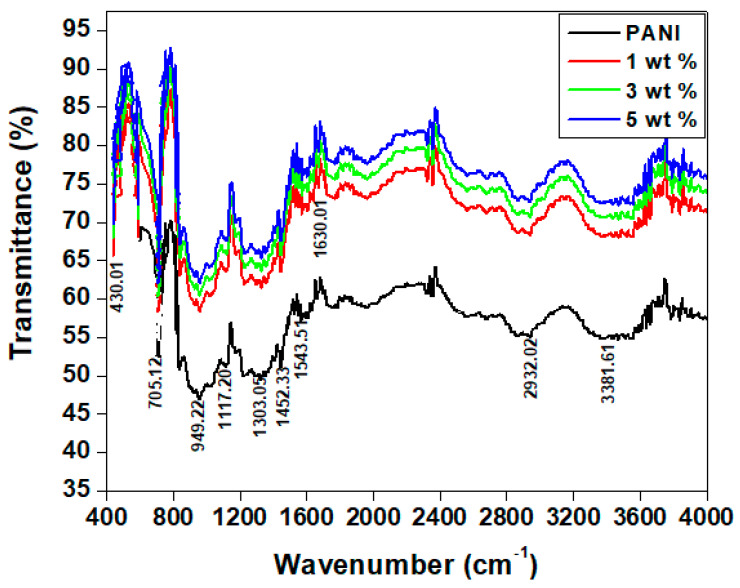
FTIR spectra of PANI and PANI—TiO_2_ nanocomposites.

The functional group of the polyaniline (PANI) and PANI—TiO_2_ nanocomposites with various weight percentages were studied by FTIR spectra, as shown in Figure 1. The characteristic peaks of polyaniline observed at 705.12 cm^−1^ are attributed to C—H bending vibration asymmetry out of the plane, 949.22 cm^−1^ is due to the p-disubstituted benzene intermediates formed during the polymerization of the aniline, 1117.20 cm^−1^ is the characteristic peak of conductive polyaniline and is due to the charge delocalization on the polymer backbone, 1303.05 cm^−1^ corresponds to the stretching of C—N of the aromatic ring, 1452.33 cm^−1^ is due to C=N vibration of the benzenoid ring, 1543.51 cm^−1^ is due to the stretching of C=C of the quinoid ring (N=Q=N), 2932.02 cm^−1^ is due to the stretching vibrations N–H of the secondary amine, and 3381.61 cm^−1^ is due to the bending vibration of the hydroxyl group (O—H) of water molecules [36,37,38]. In the TiO_2_-doped PANI nanocomposites, the characteristic peaks of polyaniline are indicated at 705.12 cm^−1^, 949.22 cm^−1^, 1117.20 cm^−1^, 1303.05 cm^−1^, 1452.33 cm^−1^, 1543.51 cm^−1^, 2932.02 cm^−1^, and 3381.61 cm^−1^. In addition, the nanocomposites show two other characteristic peaks for TiO_2_, at 430.01 cm^−1^, which corresponds to the vibration of Ti—O—Ti along with the plane, and at 1630 cm^−1^, which corresponds to Ti –OH stretching in the plane [39,40,41].

### 4.2. XRD Pattern

**Figure 2:**
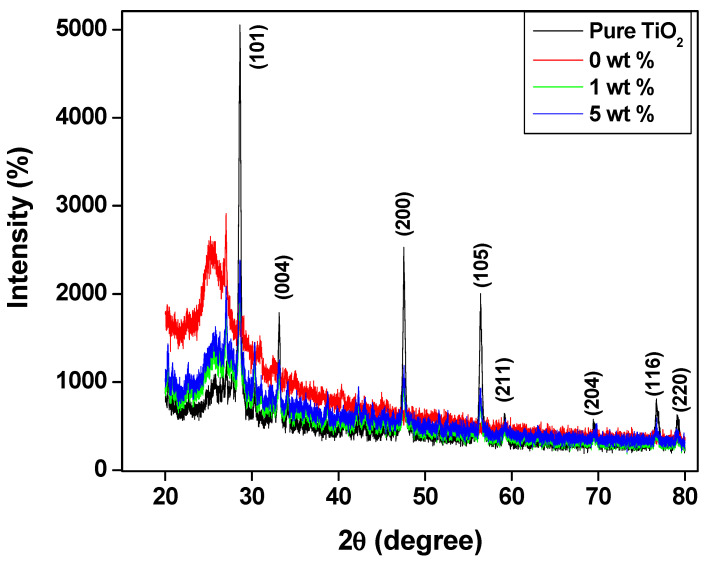
XRD pattern of PANI and PANI—TiO_2_ nanocomposites.

Figure 2 shows the X-ray diffraction (XRD) pattern of the pure TiO_2_ and the PANI—TiO_2_ nanocomposites of different weight percentages. The XRD pattern of the TiO_2_ indicates the anatase phase, with a tetragonal structure vide ref JCPDS 21–1276 with diffraction 2θ peaks at 28.9°, 32.4°, 48.8°, 56.2°, 59.2°, 69.8°, 77.4°, and 79.6°, which correspond to the hkl value of the (101), (004), (200), (105), (211), (204), (116), and (220) planes. The size of the TiO_2_ was calculated using the Debye Scherer equation and was found to be 12 nm. These important characteristic peaks appeared in the PANI—TiO_2_ nanocomposite XRD pattern along with the semicrystalline peak of polyaniline at 26.9° and 29.3° because of the intermolecular polyaniline chain [42]. 

Because of the presence of benzenoid and quinonoid groups in the polyaniline, the polymer is semicrystalline in form. Bragg’s Law and the Debye Scherer equation were used to calculate the interplanar crystallinity distance and crystal size [43].

$$D=\frac{k\lambda }{\beta Cos\theta }$$

where D is the crystalline size of the nanoparticles, *K* is the Scherrer constant (0.98), marks the wavelength (1.54), and signifies the full width at half maximum (FWHM).

### 4.3. SEM Analysis

**Figure 3:**
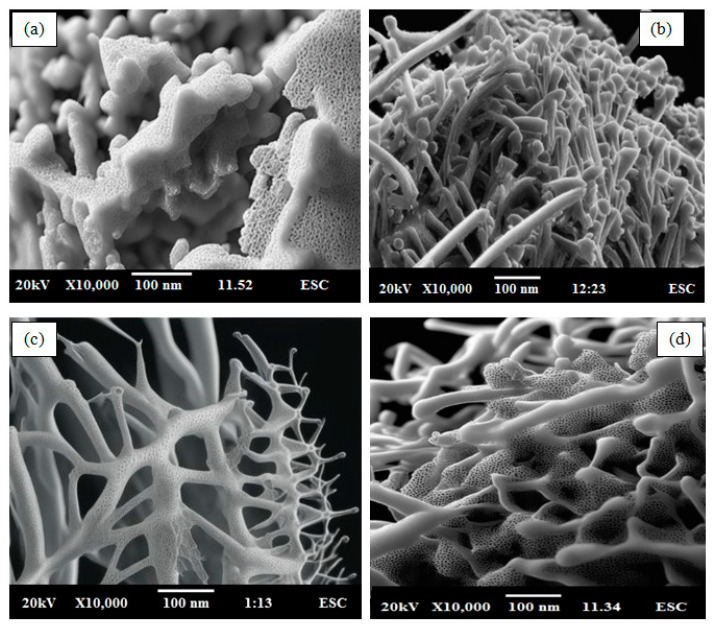
SEM image of (**a**) TiO_2,_ (**b**) PANI, (**c**) 3 wt.%, and (**d**) 5 wt.% PANI—TiO_2_ nanocomposites.

The enhancement of hydrogen storage is significant in polymer fibers doped with porous metal oxide compared to bulk materials due to the high surface-to-volume ratio. It is found that the titanium precursor in the soft template array developed well-defined highly porous TiO_2_ nanoparticles with a pore size of less than 2 nm and a formed grain around 60nm, as shown in Figure 3a. It is observed that the polyaniline prepared in an ice bath by the oxidation polymerization method using an anionic surfactant of ammonium lauryl sulfate helps to create capillary polymerization in ice and, as a result, long polyaniline fibers are formed around 100 nm, as shown in Figure 3b. Further, it is observed that when below 3 wt.% TiO_2_ nanoparticles are dispersed in an aniline mixture during the in situ polymerization, the nanoparticles are completely embedded or covered with the polyaniline without any nanoparticles agglomeration during polymerization, as shown in Figure 3c. This well-controlled in situ polymerization method in the presence of anionic surfactant assists in designing the required TiO_2_-doped polyaniline nanocomposite. However, it is noted that the doping of TiO_2_ in polyaniline above 3 wt.% significantly distorted the formation of polyaniline nanocomposite fibers, as shown in Figure 3d. The 5 wt.% PANI—TiO_2_ nanocomposites shows a fiber size of around 88 nm. Further, it is also noted that the polyaniline nanocomposites are highly porous in nature and, as a result, they exhibit favorable hydrogen storage application [44,45,46].

### 4.4. DC Conductivity

**Figure 4:**
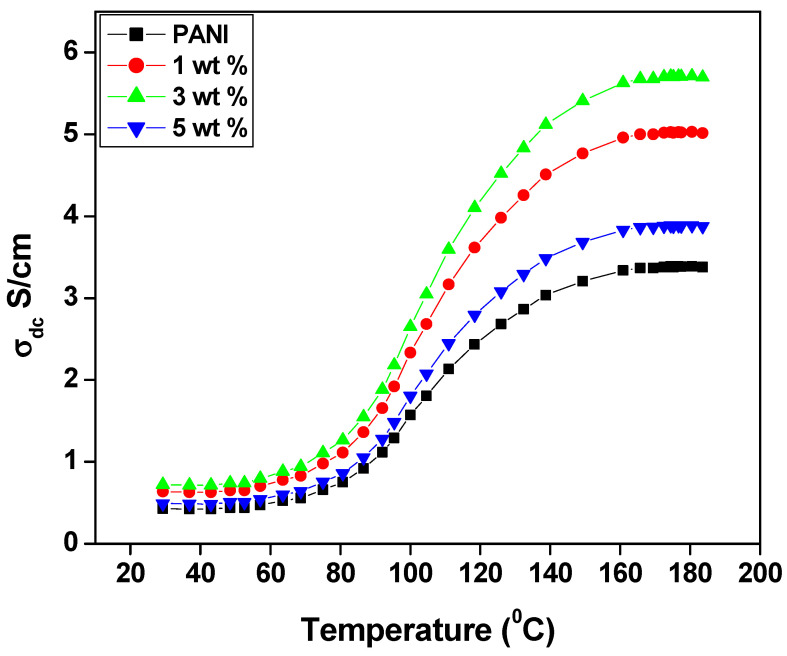
DC conductivity of PANI and PANI—TiO_2_ nanocomposites.

Figure 4 shows the DC conductivity of the polyaniline and PANI—TiO_2_ nanocomposites as a function of temperature. It is observed that the conductivity of the polyaniline and its nanocomposites increases with an increase in temperature from 25 °C to 190 °C; this may be due to the increases in the charge carriers. An increase in DC conductivity as a function of an increase in temperature causes a decrease in activation energy (Ea) and is associated with a shift of the Fermi level in the doped TiO_2_ nanocomposites. The temperature dependence of the conductivity of the nanocomposites exhibits a typical semiconductor behavior and can be expressed by the one-dimensional variable-range hopping (1D-VRH) model proposed by Mott and Davis as follows;

$$\rho \left(T\right)=\rho_{0}\;exp\left[{\left(\frac{T_{0}}{T}\right)}^{1/p}\right]$$

where the pre-exponential factor $\rho_{0}$ depends only on lower temperature *T*, T_0_ is the characteristic hopping temperature, and *p* is the exponential factor. According to Mott and Davis, a $\rho_{0}$ DC conductivity value suggests that conduction occurs primarily in extended states [47,48]. A lower value *ρ*_0_ suggests a large range of localized states, and conduction occurs via the hopping of charge carriers from balance bands to conduction bands. Because of the large variety of localized states present in the nanocomposites, the values of so in the polyaniline fiber structure are of the order of 5–6 S/cm for PANI and 3 wt.% of PANI/TiO_2_ nanocomposites, implying that conduction occurs via the hopping process. Based on the findings, we can deduce that the hopping mechanism causes the rise in nanocomposite conductivity [49,50]. Polarons and bipolaron creation can also be utilized to describe the conduction mechanism. Polarons and bipolarons have a significant impact on the charge injection in the transport properties of polyaniline nanocomposites [51]. These are self-localized defects associated with distinctive polyaniline backbone distortions and quantum states deep in the energy gap due to strong lattice coupling. 

The conductivity of the polyaniline nanocomposites shows three steps of conductivity along with the temperature change. In the first step, the temperature range from 20 °C to 80 °C is almost constant, which may be due to insufficient energy to move the polarons and bipolarons from a low energy level to a higher energy state. In the second step, the temperature range of 80 °C to 120 °C shows a gradual increase in conductivity, which is due to the mobility of the electron charge system, and in the third step, the exponential increase in conductivity observed from the range of 120 °C to 190 °C may be due to the hopping of polarons and bipolarons from the ground state to the excited state, which is the typical behavior of polyaniline semiconductors. The maximum conductivity was found to be 5.6 S/cm for 3 wt.% of polyaniline—TiO_2_ nanocomposite [52].

### 4.5. Cyclic Voltammetry

A polyaniline-coated polyester sheet was used to make a working electrode. The polyaniline and its nanocomposites were used as a conducting substance and polystyrene (PS) was used as a coating support material. Polyaniline cannot produce a covering with suitable mechanical characteristics on its own. As a result, polystyrene was employed as a support material to create a covering containing conducting polyaniline. The coating solution was prepared and deposition was carried out in the following manner. First, 20 mL of chloroform was used to dissolve 1.00 g of polyaniline and 1.27 g of camphorsulfonic acid (C_10_H_16_O_4_S) (CSA). The solution was then treated with 1.00 g of polystyrene. To create the coating solution, the solution was magnetically swirled for 24 h. A chloroform solution of polyaniline and camphorsulfonic acid was produced and a polystyrene solution was added to it. The resultant solution was used to prepare a polymer solution for the coating. The polyaniline was combined with camphorsulfonic acid to form a PANI-CSA salt which was mixed in a molar ratio of 2:1, with the polyaniline computed using the aniline monomeric unit. The salt of polyaniline and CSA was easily soluble in chloroform at this ratio. Because this mixture had sufficient conductivity, the PANI: PS weight ratio utilized in this experimental work was 1:1 [53,54,55].

**Figure 5:**
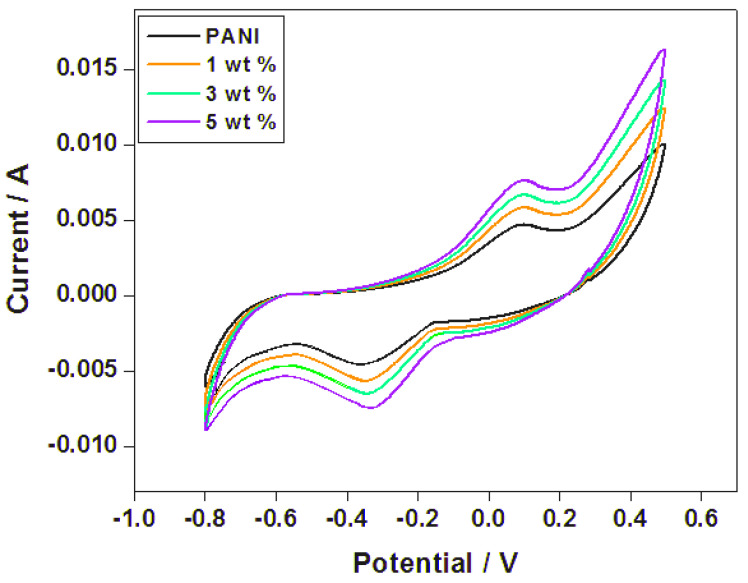
Cyclic voltammetry (CV) of PANI and PANI−TiO_2_ nanocomposites.

In Figure 5, the cyclic voltammetry (CV) curves of the PANI and PANI—TiO_2_ nanocomposite display a generally rectangular shape, indicating the electric double layer capacitance (EPLC) feature. The CV curve of PANI, on the other hand, reveals the usual CV characteristic of PANI, attributed to the redox peaks of 0.93 V and 0.24 V. The two peaks are due to PANI’s redox transition from a semiconducting to a conducting state (polaronic emeraldine form) and the transformation of emeraldine–pernigraniline [56]. 

### 4.6. Hydrogen Sorption Measurement

Volumetric hydrogen sorption measurements are critical for understanding PANI and PANI—TiO_2_ nanocomposite fibers for hydrogen storage behavior. At 60 °C, hydrogen absorption was carried out at a high pressure of 80 bar in a pre-calibrated reservoir. Hy-Energy’s PCTPro 2000 sorption machine was used for the isothermal volumetric analyses. Sievert’s equipment is fully automated and has an internal PID-controlled pressure regulator with a pressure of 170 bars. The HyDataV2.1 Lab-View program was used to examine the software subroutines for hydrogen purging cycles, leak testing, and PCT [57,58].

**Figure 6:**
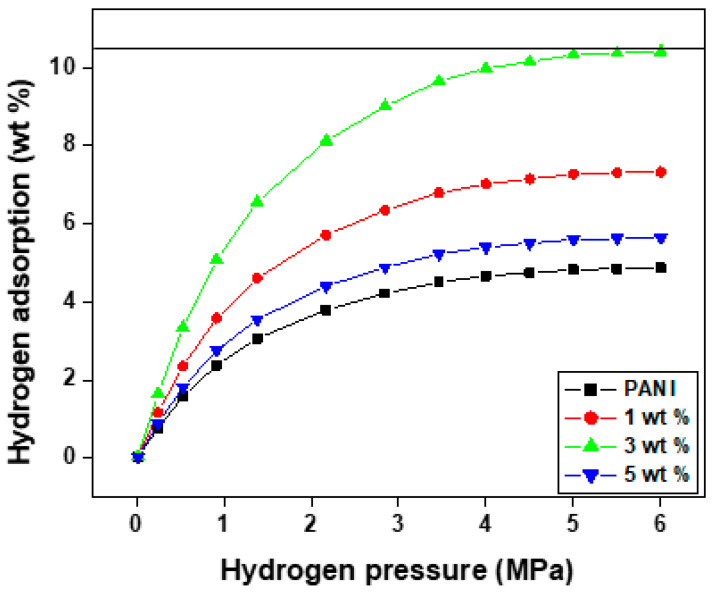
Hydrogen adsorption of PANI and PANI—TiO_2_ nanocomposites.

The anionic surfactant of the ammonium lauryl sulfate-based polyaniline and the PANI—TiO_2_ nanocomposite fibers were placed in a Schlenk flask, which was covered with a rubber cap and dried under vacuum at 70 °C for 60 min under a dry vacuum. The polyaniline and its nanocomposites were completely dried before being transported to a high-pressure hydrogen reactor in a glove box under inert nitrogen gas. To investigate the sorption performance, the completely sealed reactor was linked to a high hydrogen pressure volumetric set-up. Figure 6 depicts the fluctuation of hydrogen absorption as a function of applied pressure at room temperature. It has been discovered that, when pressure increases, the capacity of hydrogen adsorption increases. At room temperature, polyaniline fiber has a hydrogen adsorption capacity of about 4.5 wt.%, but at 60 °C its capacity doubles to around 7.3 wt.%. Conversely, 3 wt.% of PANI—TiO_2_ nanocomposites show a high absorption capacity of 10.4 wt.% compared with other nanocomposites, which may be due to the particular architecture and shape, indicating that the fiber is porous in nature and suggesting the nitrogen (N) molecules in the polyaniline are the donor of the hydrogen bond and a large number of hydrogen bond receptor sites exist in the molecular structure [59]. Hydrogen adsorption occurs in two ways. First, the polyaniline backbone has two receptors in the conductive groups that increase relative to the quinoid diimine units (N=B=N), resulting in an increased electronic transition that interacts with the hydrogen atoms. The H-H bond breaks through the heterolytic route to produce a hydride, bonding to the Ti site, and proton bonds to one oxygen atom to generate the (H+-H) species in the second phase, the physisorption of H_2_ molecules (H_2_* species). Later, the H on the Ti^4+^ → Ti^3+^ site is transferred to the neighboring O site, resulting in homogenous dissociation and the formation of 2O-H hydroxyl groups labeled (H+-H+). This step is accompanied by a two-electron transfer to surface titanium sites that become reduced [60].

**Figure 7:**
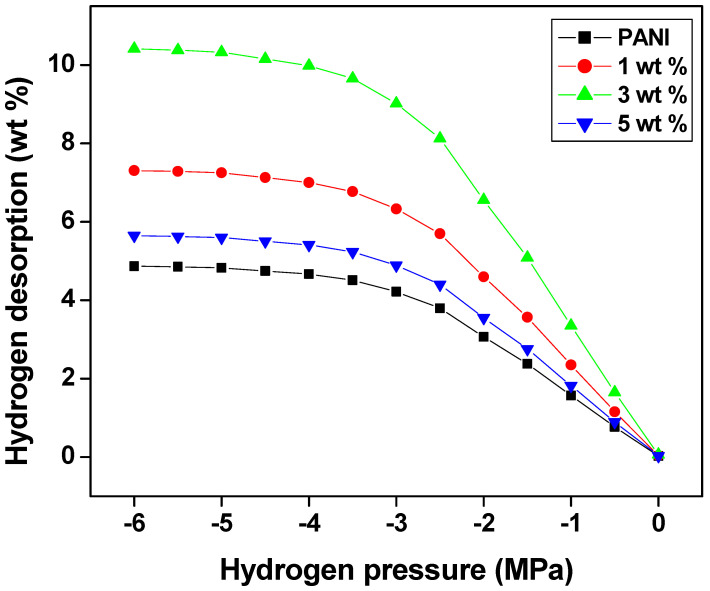
Hydrogen desorption of PANI and PANI—TiO_2_ nanocomposites at 60 °C.

Figure 7 shows the hydrogen desorption of PANI and PANI—TiO_2_ nanocomposites at 60 °C as a function of applied pressure. It is observed that hydrogen desorption increases with the release of pressure from 10.4 wt.% to 0 wt.% by an increase in temperature from room temperature to 60 °C. However, it is also important to note that the desorption process is very slow at room temperature which may be due to the strong N=H bonding of the polyaniline backbone molecules. The desorption of hydrogen at room temperature for the polyaniline and PANI—TiO_2_ nanocomposites occurs at 78% and 86% for 3 wt.%, respectively [61]. 

**Figure 8:**
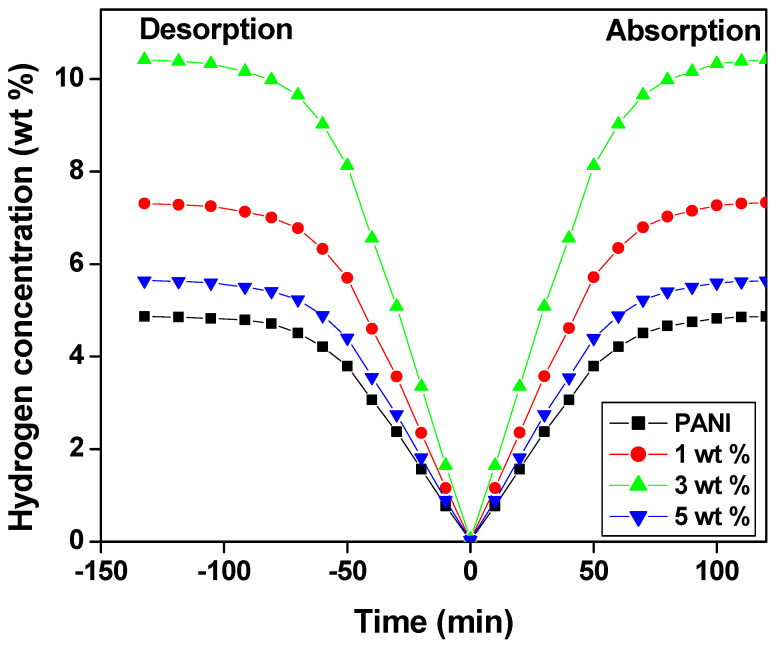
Hydrogen absorption/desorption as a function of time.

The initial hydrogen uptake of the polyaniline and PANI—TiO_2_ nanocomposite fibers occurred at room temperature and prepared by in situ polymerization in an ice bath over a period of time, as shown in Figure 8. It is realized that polyaniline nanocomposite fiber absorption has reached saturation in less than 80 min, i.e., 88% of the real capacity of 10.4 wt.%. Furthermore, when the temperature is increased up to 60 °C at the pressure of 1 bar, roughly 2–4 wt.% of hydrogen absorption occurs at the optimal temperature by the polyaniline nanocomposite fibers. However, the hydrogen gas desorption requires a longer period of 140 min, which may be due to the strong N=H bonding of the polyaniline backbone molecules and the hygroscopic nature of TiO_2_ nanoparticles [62]. The pressure–composition–temperature (PCT) study for the polyaniline fiber was performed at room temperature to establish the efficiency of the hydrogen sorption. A separate variable pressure forms an effective hydride around 6 bar after 80 min. It exponentially decreases up to 90 wt.% hydrogen uptakes with a linear zone of 4.5 wt.%. An overall desorption capacity of 10.4 wt.% reduced to 96% was found for 3 wt.% TiO_2_-doped PANI nanocomposites [63,64]. 

## 5. Conclusions

Polyaniline and its nanocomposite fibers prepared by porous metal oxide exhibit excellent hydrogen storage applications. In this study, polyaniline and porous TiO_2_-doped polyaniline nanocomposite fibers were prepared using the in situ polymerization technique in the presence of ammonium lauryl sulfate anionic surfactant. The synthesized polyaniline nanocomposites were characterized by FTIR spectroscopy and XRD pattern for structural analysis. The FTIR spectra show the characteristic peak of the benzenoid ring for C-N stretching and the quinoid ring of C-H bending motion, along with the metal oxide peaks, confirming the formation of TiO_2_ in the polyaniline nanocomposites. The XRD pattern shows the semicrystalline nature of the polyaniline and TiO_2_, indicating the rutile phase with a tetragonal structure in the nanocomposites. The surface morphology was studied by SEM images, confirming the formation of the fiber structure embedded with porous TiO_2_ nanoparticles. DC conductivity shows the three steps of conductivity, consistent with a semiconductor. Among all the nanocomposites, the maximum DC conductivity was found to be 5.6 S/cm for 3 wt.% polyaniline–TiO_2_ nanocomposite. Cyclic voltammetry shows the characteristics of the PANI, attributed to the redox peaks 0.93 V and 0.24 V. The two peaks are due to the PANI’s redox transition from a semiconducting to a conducting state. The hydrogen absorption capacity is approximately 4.5 wt.%, but at 60 °C the capacity doubles to approximately 7.3 wt.%. Conversely, 3 wt.% PANI—TiO_2_ nanocomposites have a high absorption capacity of 10.4 wt.% compared to the other nanocomposites. An overall desorption capacity of 10.4 wt.% reduced to 96% was found for 3 wt.% TiO_2_-doped PANI nanocomposites. Hence, it is evident that polyaniline nanocomposite fibers are promising candidates for hydrogen storage applications.

## Data Availability

The data presented in this study are available on request from the corresponding author.
